# CohesinDB: a comprehensive database for decoding cohesin-related epigenomes, 3D genomes and transcriptomes in human cells

**DOI:** 10.1093/nar/gkac795

**Published:** 2022-09-27

**Authors:** Jiankang Wang, Ryuichiro Nakato

**Affiliations:** Institute for Quantitative Biosciences, The University of Tokyo, Bunkyo-ku, Tokyo, Yayoi 1-1-1, Japan; Graduate School of Medicine, The University of Tokyo, Bunkyo-ku, Tokyo, Hongo 7-3-1, Japan; Institute for Quantitative Biosciences, The University of Tokyo, Bunkyo-ku, Tokyo, Yayoi 1-1-1, Japan; Graduate School of Medicine, The University of Tokyo, Bunkyo-ku, Tokyo, Hongo 7-3-1, Japan

## Abstract

Cohesin is a multifunctional protein responsible for transcriptional regulation and chromatin organization. Cohesin binds to chromatin at tens of thousands of distinct sites in a conserved or tissue-specific manner, whereas the function of cohesin varies greatly depending on the epigenetic properties of specific chromatin loci. Cohesin also extensively mediates cis-regulatory modules (CRMs) and chromatin loops. Even though next-generation sequencing technologies have provided a wealth of information on different aspects of cohesin, the integration and exploration of the resultant massive cohesin datasets are not straightforward. Here, we present CohesinDB (https://cohesindb.iqb.u-tokyo.ac.jp), a comprehensive multiomics cohesin database in human cells. CohesinDB includes 2043 epigenomics, transcriptomics and 3D genomics datasets from 530 studies involving 176 cell types. By integrating these large-scale data, CohesinDB summarizes three types of ‘cohesin objects’: 751 590 cohesin binding sites, 957 868 cohesin-related chromatin loops and 2 229 500 cohesin-related CRMs. Each cohesin object is annotated with locus, cell type, classification, function, 3D genomics and cis-regulatory information. CohesinDB features a user-friendly interface for browsing, searching, analyzing, visualizing and downloading the desired information. CohesinDB contributes a valuable resource for all researchers studying cohesin, epigenomics, transcriptional regulation and chromatin organization.

## INTRODUCTION

Cohesin is an essential protein complex that encircles chromatin within its ring-shaped structure (1). While the function of cohesin was initially discovered in relation to the cohesion of duplicated sister chromatids during mitosis (2), modern molecular biology has identified cohesin as a crucial factor in transcriptional regulation and chromatin structure. Cohesin is present in almost all active enhancer regions (3). Cohesin has the ability to block enhancer-promoter interactions with CCCTC-binding factor (CTCF) (4) or mediate cis-regulatory modules (CRMs; genomic regions in which multiple transcription factors bind to regulate gene expression) in the absence of CTCF (5). In mammalian cells, cohesin plays a role in maintaining chromatin organization, including chromatin loops and topologically associated domains (TADs) (6,7). An abnormal state of cohesin impacts the precise chromatin organization, which then leads to the pathogenic phenotypes in a wide range of human diseases (8,9), including the developmental disorder Cornelia de Lange syndrome (CdLS) (10) and multiple types of cancers (11). The extensive roles of cohesin have attracted the attention of many molecular biologists, especially those studying transcriptional regulation and chromatin structure.

Within the past few decades, advances in next-generation sequencing (NGS) technologies have enabled the genome-wide analysis of cohesin from different aspects. However, the integration and exploration of the massive cohesin datasets are not straightforward. First, the chromatin binding sites of cohesin are highly diverse. Unlike typical transcription factors (TFs) or CTCF, cohesin does not show any binding motifs, impeding the computational prediction of cohesin sites. Cohesin can bind to chromatin at tens of thousands of distinct sites in a conserved or tissue-specific manner (5,12), whereas the functions of cohesin vary greatly depending on the epigenetic properties of specific chromatin loci (i.e. context specific). For example, some cohesin is located at the boundaries of TADs where it forms a conserved architecture, while other cohesin is located inside TADs (13); some cohesin co-localizes with CTCF, while non-CTCF cohesin exists in a tissue-specific manner (5); cohesin located in promoter, intergenic or intragenic regions may also have distinct functions (14). Second, from a 3D genomic perspective, cohesin mediates chromatin interactions (15), forming different levels of organization in the nucleus. Instead of considering chromosomes as linear coordinates, the spatial organization of cohesin-mediated loops is now becoming indispensable in molecular biology (16). Third, cohesin is extensively present in CRMs (1,5). Discovering the grammar of CRMs (e.g. finding distal regulatory sites for genes of interest) is a longstanding goal of biomedical research (17). Considering the widely accepted view that cohesin is a direct regulator of gene expression (1), information on cohesin-mediated CRMs is useful for many studies. Taken together, big data on cohesin binding, cohesin function, and cohesin-mediated chromatin loops and CRMs are essential not only for the cohesin research field but for all studies of transcriptional regulation and 3D genomics. There is great demand to create a database to summarize cohesin-related information obtained from multiomics datasets.

Here, we present CohesinDB (https://cohesindb.iqb.u-tokyo.ac.jp), a comprehensive multiomics cohesin database in human cells that integrates 2,043 ChIP-seq, ChIA-PET, Hi-C, Hi-ChIP, RNA-seq and microarray datasets from 530 studies (176 cell types). CohesinDB offers a sophisticated interface for browsing, searching, visualizing and analyzing cohesin datasets and three types of ‘cohesin objects’: cohesin binding, cohesin-related chromatin loops and CRMs. We examined the reliability of the cohesin information in CohesinDB by comparing it with known knowledge. We also compared CohesinDB with related databases. CohesinDB contributes a valuable resource not only for cohesin research but also for research on various genomic events, including chromatin folding, enhancer activity, CRMs, epigenetics, transcriptional regulation, and human diseases.

## MATERIALS AND METHODS

### Data collection

**Figure 1.**
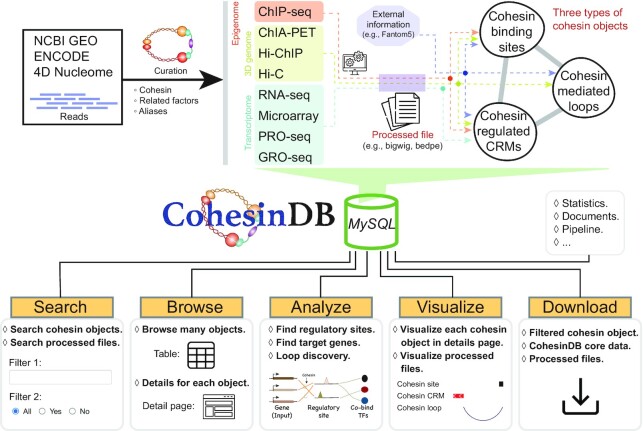
Flowchart of the construction of CohesinDB. CohesinDB collects and processes large-scale sequencing data to obtain three types of cohesin objects. This big data is then used to implement the sophisticated web interfaces.

Figure 1 shows a flowchart of the construction of CohesinDB. The raw sequencing data in CohesinDB were downloaded from the NCBI GEO/SRA (18), ENCODE (19) and 4DN projects (20). All datasets were obtained with the version of October 1, 2021. For cohesin binding sites, because cohesin consists of multiple subunits, we searched ChIP-seq data for all possible names of cohesin subunits (Rad21, SMC1, SMC3, SA1, SA2), related factors (NIPBL, Mau2, WAPL, PDS5, ESCO1, ESCO2, etc.), and aliases (e.g. the alias of SA1 could be STAG1 or SCC3). CTCF ChIP-seq data, if available in cohesin ChIP-seq studies, were also downloaded for the annotation of cohesin sites. After manual curation, we collected 962 ChIP-seq samples from 91 cell types derived from 137 studies. To obtain cohesin-mediated chromatin loops, we collected 119 cohesin-targeted ChIA-PET samples (45 cell types, 57 studies) and 42 cohesin-targeted Hi-ChIP samples (10 cell types, 11 studies). We also included 385 Hi-C samples (60 cell types, 294 studies) because although Hi-C does not target cohesin, the chromatin loops detected from Hi-C are fundamentally mediated by cohesin (7). Hi-C also provides information on chromosome structures, such as chromatin loops, compartments, TAD boundaries and chromatin hubs (21). Lastly, we collected 535 transcriptomics samples (e.g. RNA-seq, microarray) which compare gene expression levels between cohesin knockdown/knockout and control groups (71 pairs of comparisons in CohesinDB).

### Processing of sequencing data

All sequencing data were aligned to human genome build GRCh38. ChIP-seq reads (single-end or paired-end) were aligned with Bowtie2 version 2.4.1 (22), followed by peak calling with MACS2 version 2.2.6 (23) with the default parameters. Quality assessment was performed by using SSP version 1.2.2 (24). Read normalization and ‘bigwig’ file generation were performed using DROMPAplus version 1.4.0 (25). Mango version 1.2.1 (26) was used to process the ChIA-PET data. ChIA-PET data with tagmentation libraries and others were distinguished. For Hi-C and Hi-ChIP data, Juicer version 1.5.7 was used to generate contact matrices, and Juicertools version 1.11.04 (27) was used to detect chromatin loops. One-dimensional metrics (e.g. eigenvectors identifying compartments A and B) derived from Hi-C were calculated by using HiC1Dmetrics version 0.2.1 (21). Microarray data were processed using GEO2R (https://www.ncbi.nlm.nih.gov/geo/geo2r/). RNA-seq reads were mapped with STAR version 2.7.3 (28), and gene expression levels were measured by using RSEM version 1.3.1 (29). Differential gene expression was identified with edgeR version 3.34.1 (30). All of these processed data were used to construct the three types of cohesin objects (Figure 1).

### Cohesin binding sites

Only high-quality ChIP-seq data, defined according to a normalized strand coefficient (NSC) > 1.2 and number of peaks > 1000, were retained to construct cohesin binding information. After excluding indirect factors (e.g. CTCF, ESCO2, WAPL), we used 550 ChIP-seq datasets to generate the universal binding sites of cohesin. In total, we obtained 34 534 315 independent cohesin sites. We then retained cohesin sites identified in more than two ChIP-seq samples to increase the reliability of binding sites. Two sites at a distance of <200 bp were merged to avoid clustered small peaks. Finally, we obtained 751 590 cohesin binding sites (coverage 27.93% whole genome), each of which was assigned a unique ID, for the construction of CohesinDB.

### Annotations of cohesin binding sites

CohesinDB provides comprehensive annotations for each cohesin binding site (Supplementary Figure S1). (i) Basic information includes the ID, locus, data source, cell type, and nucleotide sequence. (ii) Cohesin category information includes the peak occupancy ratio (percentage of samples containing a certain cohesin site), cell-type specificity ($1 - {N}_{observed}/{N}_{all}$, where N is the number of cell types), the cohesin subunits involved (e.g. SA1), CTCF co-localization and CTCF motifs. Each site was also classified according to its intragenic, intergenic, transcriptional start site (TSS), or transcriptional end site (TES) location based on the RefSeq gene reference. (iii) For 3D genomics, each cohesin site was annotated as to whether it anchors chromatin loops detected by ChIA-PET, Hi-C or Hi-ChIP. We also utilized the Hi-C data in CohesinDB to provide annotations of TAD boundaries (31), chromatin hubs (21) and A/B compartments (27). (iv) The cis-regulatory information includes enhancers from the Fantom5 project (32), chromatin states from the Roadmap project (33), co-bound TFs from the ReMap database (34), and target genes (defined in the following section). (v) The annotations of functional elements include single-nucleotide polymorphisms (SNPs) from the GWAS Catalog project (35) (https://www.ebi.ac.uk/gwas/) and somatic mutations from the COSMIC project (36) (https://cancer.sanger.ac.uk/cosmic). (vi) Hyperlinks to the associated cohesin loop objects and cohesin CRM objects are provided. (vii) Selected cohesin sites, related cohesin loops, and target genes of related cohesin CRMs are visualized in a genome browser.

### Cohesin-related chromatin loops

Chromatin loops obtained from ChIA-PET were called by using Mango with a false discovery rate (FDR) <0.05. The loops obtained from Hi-C and Hi-ChIP were called by using HiCCUPS (27) with the default parameters. In total, we obtained 957,867 cohesin-related chromatin loops, each of which was assigned a unique ID. Supplementary Figure S2 shows the annotations of each cohesin loop: (i) basic information includes the ID, the genomic loci of the two anchors of a loop, and loop length; (ii) data source information includes the assay type (e.g. ChIA-PET or Hi-C), the cohesin subunits involved, the cell types involved, and the original study; (iii) hyperlinks to the associated cohesin binding objects and cohesin CRM objects are provided; (iv) selected cohesin loops, related cohesin sites, and target genes of related cohesin CRMs are visualized in a genome browser.

### Identification of double-evidenced cohesin-related CRMs

Cohesin-related CRMs are defined based on the pairing of cohesin sites with target genes, together with epigenomics and chromatin structural features. In CohesinDB, two types of evidence are used to identify cohesin-related CRMs. The first type, cohesin-DEGs evidence, comes from transcriptomic data in the context of cohesin depletion. Cohesin DEGs (i.e. differentially expressed genes identified after cohesin knockdown/knockout) were filtered according to a |Log_2_-fold change| >1 and FDR <0.01. Only DEGs identified in more than two studies were retained, to obtain a more reliable gene list. Then, cohesin sites less than two megabases away from the promoter of a DEG were considered regulatory sites, providing pairings between genomic loci and genes. The second type of evidence comes from cohesin-mediated loops. The pairing of cohesin sites with genes was identified based on cohesin-mediated loops that connect a cohesin site to the promoter of a gene. Cohesin sites directly located at promoters were also included in the cohesin-loop evidence. To obtain ‘double-evidenced’ CRMs, only pairs of cohesin sites with genes that appeared in both the cohesin-DEG and cohesin-loop evidence were retained. Finally, we obtained a total of 2 229 500 cohesin-related CRMs involving 15 203 target genes. Cell type information on the involved cohesin binding sites, chromatin loops and DEGs was recorded for each CRM.

### Annotations of CRMs and related genes

CohesinDB assigned an ID to each gene and annotated (Supplementary Figure S3) each gene with the following information: (i) basic information, including the gene ID, symbol, locus and gene type; (ii) CRM information, including the type of available evidence for CRMs and hyperlinks to the cohesin binding objects involved in the double-evidenced CRMs; (iii) tissue specificity, including Gini coefficients of gene expression across tissue types (raw data from the GTEx portal https://gtexportal.org) and the cell-type specificity of the regulatory cohesin sites (median value); (iv) cohesin-DEG information, including whether the gene is a cohesin DEG, the cell types involved, the original studies of cohesin-DEGs, the number of studies involved, and the cohesin subunits involved; (v) cohesin-loop information, including hyperlinks to chromatin loop objects, the loop type, the cell types involved, the original studies of the loops and the cohesin subunits involved and (vi) visualization of selected gene, related cohesin sites and cohesin loops.

### Implementation of the web database

CohesinDB was written by using Python3 with Django framework (https://djangoproject.com) version 3.0.14. All data in CohesinDB were organized and queried by using MySQL (https://mysql.com) version 8.0.28. The front-end interface was implemented by using HTML, CSS, jQuery and JavaScript. Bootstrap (https://getbootstrap.com) was used to achieve a responsive web design. CohesinDB was deployed using Ubuntu 20.04 through Nginx web server (http://nginx.org) version 1.18.0. CohesinDB is available online at https://cohesindb.iqb.u-tokyo.ac.jp without any registration or login required. CohesinDB can be used in mainstream web browsers, including Google Chrome, Mozilla Firefox, Safari and Microsoft Edge, with JavaScript enabled. The CohesinDB Django project and the data processing script are available as open-source resources at https://github.com/wangjk321/CohesinDB_public.

## DATABASE CONTENT AND USAGE

### Overall design

**Figure 2.**
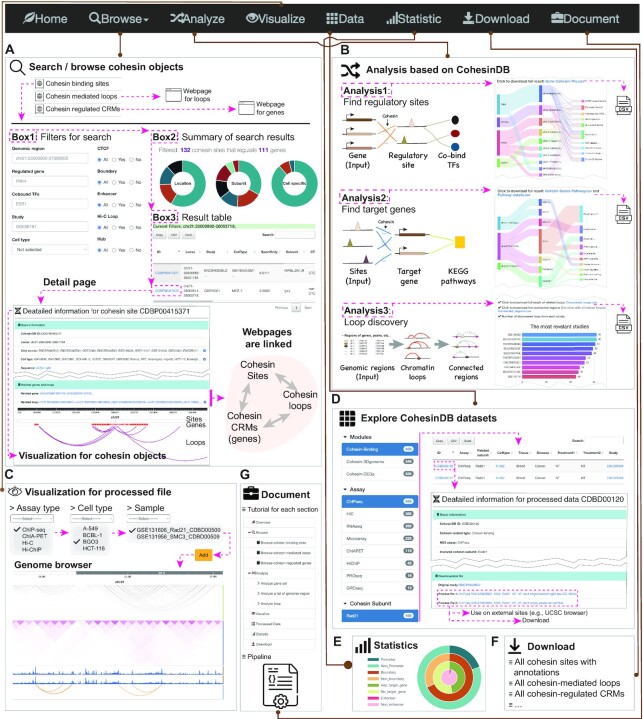
Web interface and the utilization of CohesinDB.

The contents of CohesinDB consist of four components: processed files obtained from raw sequencing data, and three types of objects concerning cohesin-binding sites, cohesin-related loops and cohesin-related CRMs (Supplementary Figure S4). Using these integrated data, CohesinDB provides a sophisticated web interface to browse, search, analyze, visualize and download the required information (Figure 2). A detailed tutorial can be found on the CohesinDB ‘document’ page (https://cohesindb.iqb.u-tokyo.ac.jp/help/).

### Browsing and searching three types of cohesin objects

The ‘Browse’ page has a drop-down list that provides browsing and searching functions for three types of cohesin objects: cohesin-binding sites, cohesin-related loops and CRMs (Figure 2A). For each type of cohesin object, we designed three boxes: a search box, where users can customize search criteria; an overview box, which summarizes the statistics; and a result box, which lists all search results. For example, in the search box for cohesin binding objects (Supplementary Figure S5), users can define the search criteria as follows: non-CTCF cohesin at chr21:25000000–27000000 in MCF-7 cells. The overview box will display the number and properties of the requested cohesin sites. The result box then allows users to check, further filter, or download cohesin binding data. By clicking on the ID of each cohesin object in the result box, users can access a detail page with annotations and visualizations (Figure 2A, Supplementary Figures S1–S3). For any of the three types of cohesin objects (e.g. a cohesin binding site), the detail pages also provide visualizations and hyperlinks for the other two types of cohesin objects (related loops and CRMs).

### Analysis of CRMs and chromatin loops

Based on the CRMs and chromatin loops in CohesinDB, we designed an analysis page with three modules (Figure 2B, Supplementary Figure S6A). The first module, ‘predict regulatory sites’, takes a gene set as input (e.g. prognostic gene signatures) and then finds all possible regulatory loci, along with the TFs at regulatory sites (Figure 2B, top, Supplementary Figure S6B). The output is displayed with a Sankey diagram, and the full results can be downloaded in a .csv file. This module exploits the strengths of transcriptomics and 3D genomics to identify gene regulatory sites, rather than just considering the distance to gene promoters (37).

The second module, ‘predict target gene’, takes a set of genomic regions as input and then discovers genes regulated by the input regions as well as the KEGG pathway enrichment of the discovered genes (Figure 2B, middle, Supplementary Figure S6C). The output includes a download of the analysis results and a Sankey diagram showing the connections between input regions, identified genes and enriched pathways. The input genomic regions can be derived from diverse sources, such as the binding sites of some TFs, differential histone modification sites, or single-nucleotide polymorphisms identified from genome-wide association studies.

The third module, ‘loop discovery’, takes a file containing genomic regions as input and then outputs chromatin loops anchored to the input region as well as the other end of the chromatin loops (Figure 2B, bottom, Supplementary Figure S6D). Users can download the matched results and examine a bar plot summarizing the most relevant samples.

Users can also specify the cell type of interest when analyzing CRMs and chromatin loops. All three modules can be tested with a ‘Run example’ button. Given the extensive involvement of cohesin in transcriptional regulation and chromatin organization, these analyses can be used for a wide range of purposes.

### Visualization

CohesinDB prepares visualizations of not only each cohesin object (Figure 2A) but also all processed data (Figure 2C). CohesinDB uses an embedding of the WashU epigenome browser (38) to visualize ChIP-seq, ChIA-PET, Hi-ChIP and Hi-C data. On the ‘Visualize’ page, users can create a multiomics visualization in the genome browser (Supplementary Figure S7) by selecting data of interest through a series of drop-down lists. The data URLs displayed in each track can also be used elsewhere, such as the UCSC genome browser (https://genome.ucsc.edu/). Visualizations in CohesinDB can be downloaded as PDF files.

### Data, download and other web pages

All processed data from the 2043 samples are listed on the ‘Data’ page (Figure 2D, Supplementary Figure S8A). This page is designed for users who want to check dataset information or reanalyze processed files (e.g. bigwig files of cohesin ChIP-seq). Users can filter datasets on the left panel, explore the result table on the right, and access the detail page by clicking on the ID (Supplementary Figure S8B) or cell name (Supplementary Figure S8C). The download page (Figure 2F) includes the core data used in CohesinDB, such as all annotated cohesin sites with annotations. In addition, the ‘Statistics’ page shows a summary of all datasets and cohesin objects in CohesinDB (Figure 2E), whereas the ‘Document’ page provides a complete tutorial for CohesinDB (Figure 2G).

### Database maintenance

The contents of CohesinDB will be maintained and updated in two ways: through a Django admin page, where we can manually modify the information of each dataset and cohesin object, or a programmable pipeline, through which we can process new datasets and update all contents (https://cohesindb.iqb.u-tokyo.ac.jp/pipeline/#maintain). The web server, web domain and SSL certificate of CohesinDB are provided by an academic institution, and we anticipate that CohesinDB will be maintained for a long time.

## STATISTICS AND VERIFICATIONS

**Figure 3.**
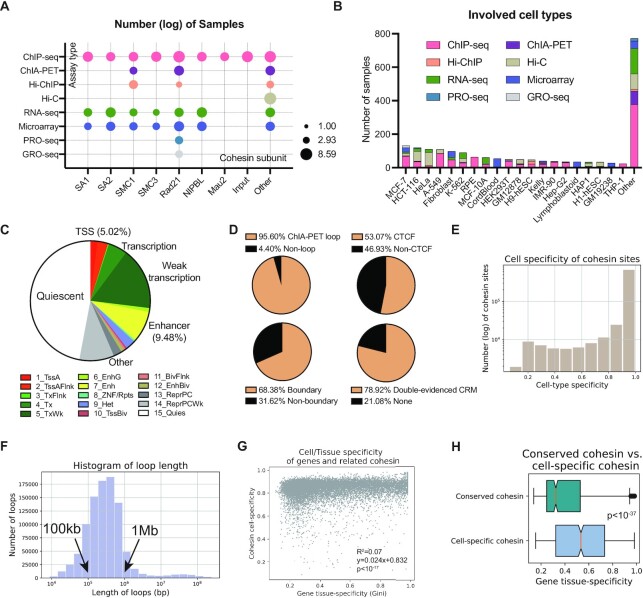
Statistics and verifications of cohesin information in CohesinDB. (**A**) Numbers of samples used for each NGS assay and each cohesin subunit. (**B**) Numbers of samples for the involved cell types. (**C**) Chromatin state annotation of all cohesin sites in CohesinDB. (**D**) Other classifications of cohesin sites. (**E**) Histogram of cell-type specificity for all cohesin sites. 0: conserved; 1: cell type specific. (**F**) Length distribution of all cohesin loops in CohesinDB. (**G**) Correlation between the cell type specificity of the cohesin site and the tissue specificity of its regulated genes based on the CRMs in CohesinDB. (**H**) Box plot showing that cell-type-specific (lower quartile) cohesin sites tend to regulate tissue-specific genes. The P value was obtained by the Mann−Whitney *U* test.

CohesinDB was built based on 2,043 multiomics datasets including many cohesin subunits, most of which were Rad21 and SMC1 subunits (Figure 3A). CohesinDB is also a multi-cell-type database. The numbers of samples for the top 20 cell types are listed in Figure 3B, with the most involved cell types being MCF-7, HCT-116 and HeLa cells.

Cohesin plays context-specific roles depending on the specific details of chromatin loci (12). To check the different functions and classifications of cohesin, we examined annotations of cohesin binding objects in CohesinDB. Figure 3C shows the diverse cohesin functions according to chromatin state learning (33). For example, 2.63% of the cohesin sites are located in heterochromatin, 5.02% are annotated as TSSs and 9.48% are annotated as enhancers. Fantom5 data (32) also shows that 4.89% of cohesin sites overlap with enhancers (Supplementary Figure S9A). Cohesin function can vary depending on the genomic distribution (14). In CohesinDB, 38.88% of cohesin sites are intragenic, and 26.79% of sites are intergenic (Supplementary Figure S9B). Other classifications are shown in Figure 3D. Importantly, almost all cohesin binding sites (95.60%) also overlap with cohesin ChIA-PET chromatin loops. This consistency between the ChIP-seq and ChIA-PET data suggests the reliability of cohesin sites and cohesin loops in CoheinDB. In Figure 3D, 53.07% of cohesin sites show co-binding with CTCF, 68.38% of cohesin sites are located at TAD boundaries, and 78.92% of cohesin sites are involved in double-evidenced CRMs. In addition, cohesin binding sites can be conserved or cell type specific (5,14). Figure 3E summarizes the cell-type specificity of all cohesin sites in CohesinDB (0: conserved, 1: specific). Quick verification can be performed by comparing the cohesin sites with and without CTCF. Supplementary Figure S9C shows that non-CTCF cohesin sites present significantly higher cell type specificity than CTCF cohesin sites, which is consistent with previous research (5).

CohesinDB provides an atlas for cohesin-related chromatin loops (7). The length of the chromatin loops included in CohesinDB ranges from a few hundred kilobases to several megabases. Figure 3F shows that the majority (76.61%) of loops are 100 kb to 1 Mb in length, while 5.17% of the loops are greater than 2 megabases in length. This is consistent with a previous report describing loop lengths of 40 kb to 3 Mb (7).

The CRMs in CohesinDB provide connections between genomic regions and target genes. To examine these connections, we analyzed the correlation between the cell type-specificity of cohesin binding and the tissue-specific expression of genes. In Figure 3G, we do not observe a strong correlation (*R*^2^ = 0.07). This is reasonable because cohesin plays context-specific roles in transcriptional regulation (14). The weak correlation can also be explained by the complexity of CRMs: one gene can be regulated by multiple sites, and one site can regulate multiple genes. Linear regression suggested a weak positive correction (slope = 0.024, *P* < 10^−17^), indicating a tendency for cell-type specific cohesin to regulate tissue-specific genes. Indeed, conserved cohesin and cell-type-specific cohesin exhibit a significant difference (*P* < 10^−37^) in the tissue specificity of the target genes (Figure 3H). This is, for the CRMs in CohesinDB, cell type-specific cohesin tends to associate with tissue-specific genes.

CohesinDB is also designed for general purposes. To test the effectiveness of CohesinDB for non-cohesin researchers, we used CohesinDB to predict the regulatory sites of estrogen response genes, i.e. genes that are upregulated after estrogen stimulation in MCF-7 cells (RNA-seq data from GSE177045). Supplementary Figures S10A-B show that 17.78% of the predicted regulatory sites are located near TSSs, whereas the others are located distal to TSSs (maximum ∼2 Mb). This is consistent with the long-range regulation of estrogen response elements (EREs) (39). Importantly, the predicted regulatory sites are enriched with several key TFs for ERE (red color in Supplementary Figure S10C). For example, 71.04% of the predicted regulatory sites show co-binding with ESR1, which is a statistically significant difference compared to random cohesin sites or random sites (Supplementary Figure S10D). This agrees with known CRMs involved in estrogen-stimulated transcription (14).

Taken together, the statistics and examinations above support the richness and reliability of the multiomics information contained in CohesinDB.

## DISCUSSION

Since the discovery of SMC (structural maintenance of chromosomes) protein family in 1997 (40), the understanding of cohesin has been revolutionized for several times (1). Modern molecular biology has recognized cohesin as a multifunctional protein responsible for the fundamental regulation of both transcription and chromatin organization (11). By collecting massive amounts of NGS data, CohesinDB provides a biologist-friendly platform for exploring cohesin binding, cohesin functions, chromatin loops, and CRMs. While cohesin and related topics (e.g. CTCF, Hi-C) are significant research fields in molecular biology, we also designed CohesinDB for more general purposes. For example, in the ‘Analyze’ section, users can obtain possible regulatory loci for any gene set (e.g. prognostic gene signatures) or obtain the target genes for any genomic region (e.g. binding sites of TFs). The integrated multiomics resource in CohesinDB can be easily used for a variety of research topics.

Some published databases are related to certain aspects of CohesinDB (Table 1). CistromeDB (41), ChIP-Atlas (37) and ReMap (34) include several ChIP-seq datasets for cohesin binding sites. However, binding sites alone are insufficient to explore the diversity of cohesin. CohesinDB is superior in the interpretable annotation of cohesin binding, chromatin loops and CRMs. 3DIV (42) and the 3Dgenome browser (43) are databases specifically designed for the 3D genome (e.g. Hi-C data), but the chromatin interactions included in these databases are not annotated. For example, it is significant to distinguish between chromatin interactions mediated by conserved cohesin forming chromatin domains and other interactions mediated by tissue-specific cohesin linking enhancers and promoters. In contrast, the chromatin interactions in CohesinDB are integrated with epigenomics and transcriptomics. For cohesin-related CRMs, KnockTF (44) considers one cohesin knockdown RNA-seq dataset, while ChIP-Atlas (37) considers cohesin-CRMs to be genes with cohesin binding in promoters. In contrast, the cohesin-CRMs in CohesinDB are based on the combination of two types of evidence: cohesin-DEG evidence from 71 studies, and cohesin-loop evidence that considers distal promoter elements. Notably, CTCFBSDB2 (45) (*NAR* database issue, 2013) is a database designed for CTCF binding sites. Although some cohesin co-localizes with CTCF, many reports have shown that cohesin is different from CTCF (7,46). Thus, CohesinDB is the first cohesin database with integrated multiomics annotations.

**Table 1.** Comparison of CohesinDB with related database

		CohesinDB	CTCFBSDB2	CistromeDB	ChIP-Atlas	ReMap	3DIV	3Dgenome Browser	KnockTF
**Cohesin bindings**	**Peak files**	◯	×	◯	◯	◯	×	×	×
	**Binding sites**	◯	×	×	×	×	×	×	×
	**Annotations**	◯	×	×	×	×	×	×	×
**Cohesin 3D genome**	**3D genome files**	◯	×	×	×	×	◯	×	×
	**3D genome annotation (e.g. TAD)**	◯	△	×	×	×	×	◯	×
	**Chromatin loops**	◯	×	×	×	×	△	×	×
**Cohesin CRMs**	**DEGs of cohesin-KD**	◯	×	×	×	×	×	×	△
	**CRMs**	◯	×	×	△	×	×	×	×
**Others**	**CTCF data**	◯	◯	◯	◯	◯	×	×	×
	**Visualization**	◯	×	△	△	△	◯	◯	×
	**Customized analysis**	◯	×	△	◯	×	◯	×	◯
	**Last update**	2022, August	2012, Nov	2018, Nov	2021, Oct	2022, Jan	2021, Jan	2018, Oct	2020, Jan
	**Reference**	This study	(45)	(41)	(37)	(34)	(42)	(43)	(44)

◯ Yes. × No. △ Not directly

While the current version of CohesinDB aims to integrate multiomics information from various types of human cells, other studies have also revealed the key roles of cohesin in other species (47). Of note, simply collecting cohesin binding sites for many species is not the purpose of CohesinDB, whereas this kind of information can be found in some general ChIP-seq databases, such as ReMap (34) and ChIP-Atlas (37). Currently, the challenge in constructing a multiomics cohesin database for multiple species remains the amount of available data. For example, the ENCODE project provides 25 ChIP-seq and 31 ChIA-PET datasets targeting Rad21 for human cells, but only two ChIP-seq and zero ChIA-PET datasets for mouse cells. There are 71 publicly available studies that can be used to define cohesin-DEGs in human cells, but only one study is available for Drosophila. With the increasing number of NGS datasets and advances in cross-species analysis (47), CohesinDB will be updated to support other species in the future. On the other hand, the CRMs in CohesinDB were generated based on a combination of cohesin-DEGs and cohesin chromatin loops. Other state-of-the-art methods, such as deep learning, might be promising approaches for identifying the general rules of CRMs (48,49).

In summary, CohesinDB provides the most comprehensive multiomics database for cohesin in human cells. CohesinDB will facilitate all studies related to cohesin, epigenomics, chromatin organization and transcriptional regulation.

## DATA AVAILABILITY

CohesinDB can be accessed online at https://cohesindb.iqb.u-tokyo.ac.jp/. The Django project and the code to process the raw data are available in the GitHub repository (https://github.com/wangjk321/CohesinDB_public).

## Supplementary Material

gkac795_Supplemental_File
