# Supplementary material for: CohesinDB: a comprehensive database for decoding cohesin-related epigenomes, 3D genomes and transcriptomes in human cells
Source: Nucleic Acids Res. 2022 Sep 27;51(D1):D70–9. doi: 10.1093/nar/gkac795 (PMC9825609; doi:10.1093/nar/gkac795)
Supplement: gkac795_Supplemental_File [file gkac795_supplemental_file.pdf]

# Supplementary Figures S1-10

**CohesinDB: A comprehensive database for decoding cohesin-related epigenomes, 3D genomes and transcriptomes in human cells**

**Jiankang Wang et al.**

## Supplementary Figure S1

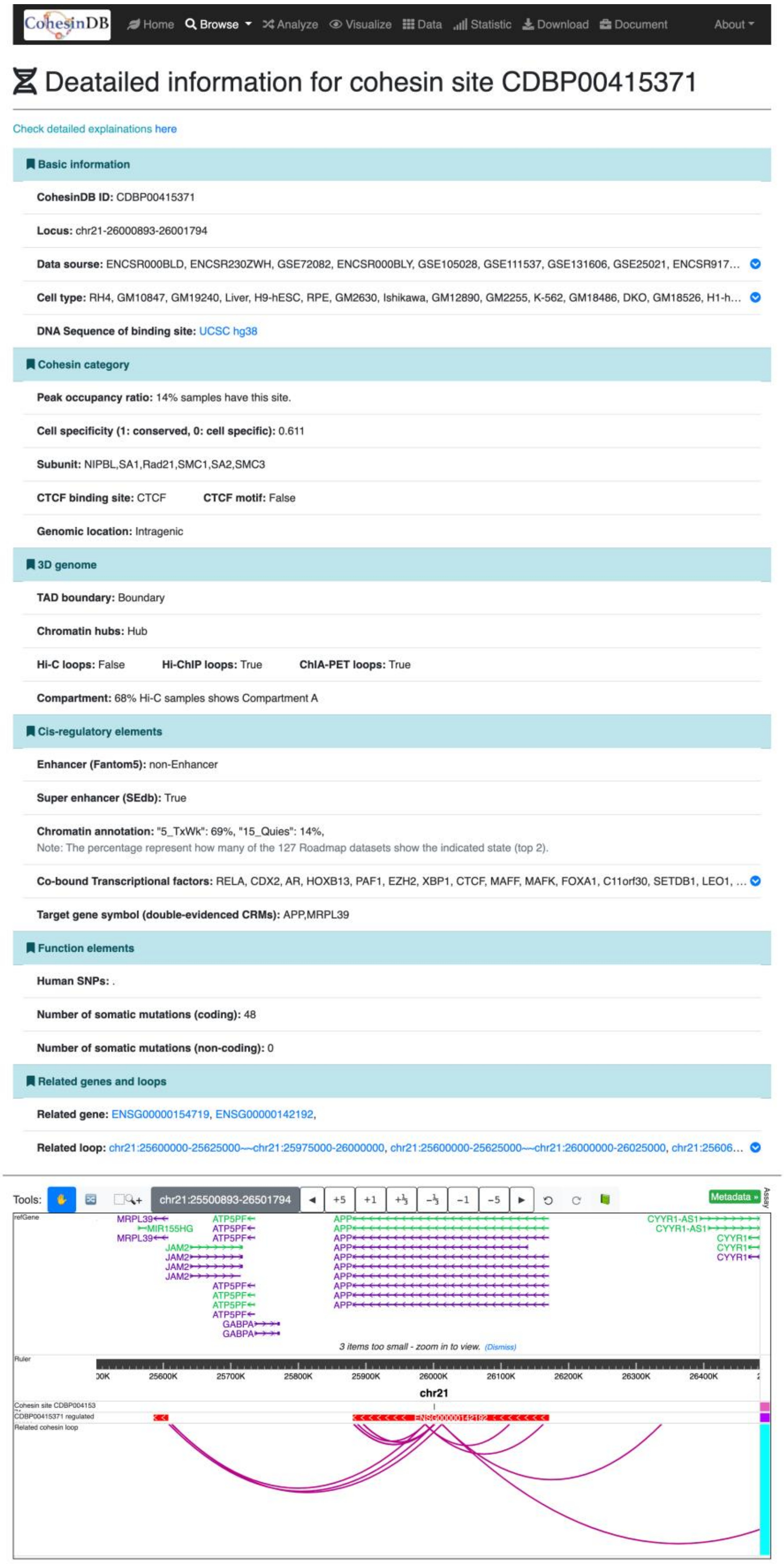


**Figure S1.** Screenshot of the detail page for a cohesin binding site object.

# Supplementary Figure S2

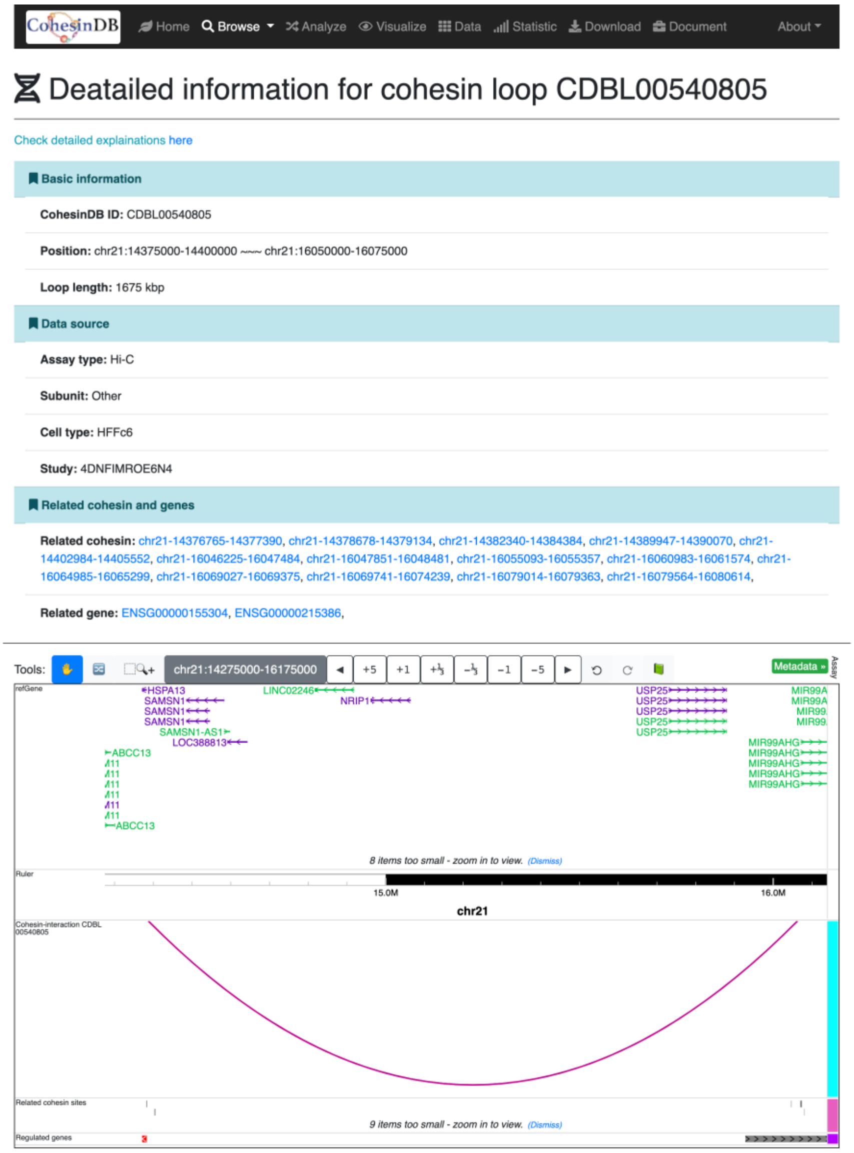


**Figure S2.** Screenshot of the detail page for a cohesin loop object.

# Supplementary Figure S3

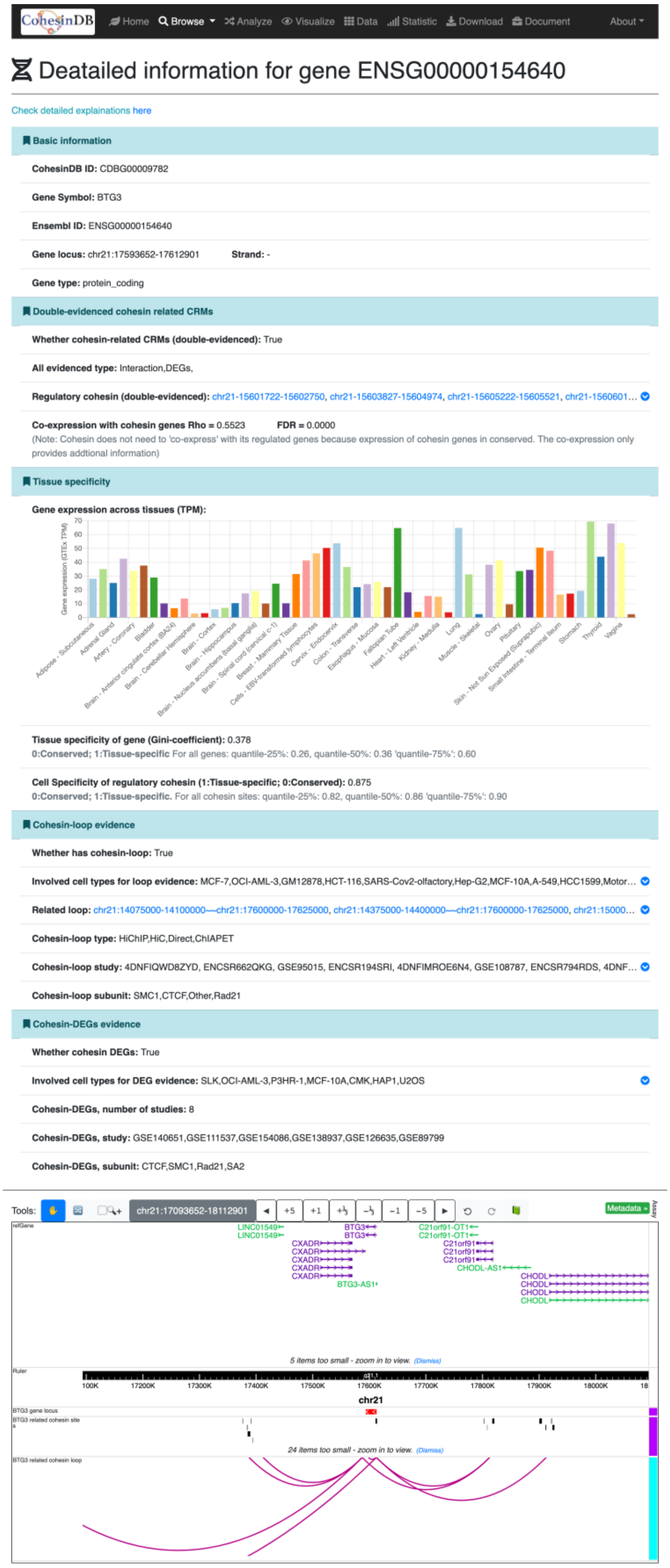


**Figure S3.** Screenshot of the detail page for a cohesin CRM object.

## Supplementary Figure S4

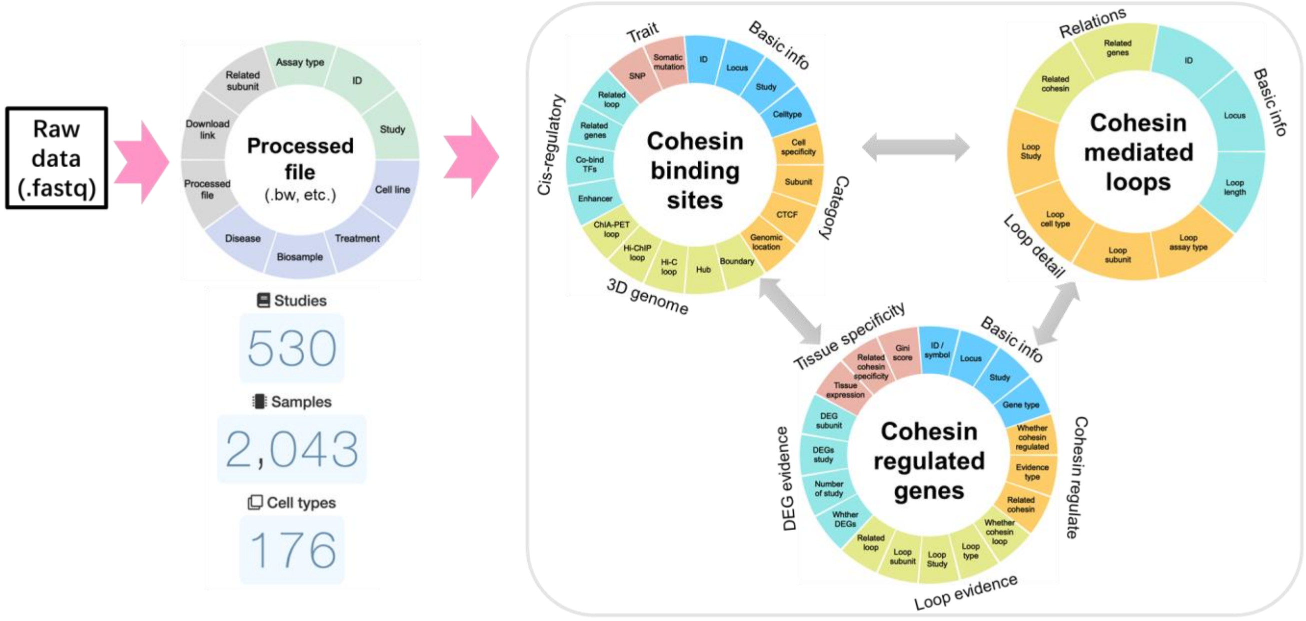


**Figure S4.** CohesinDB consists of four parts: the processed files, cohesin binding sites, cohesin-related chromatin loops and cohesin-related CRMs. The main annotations of each part are shown.

# Supplementary Figure S5

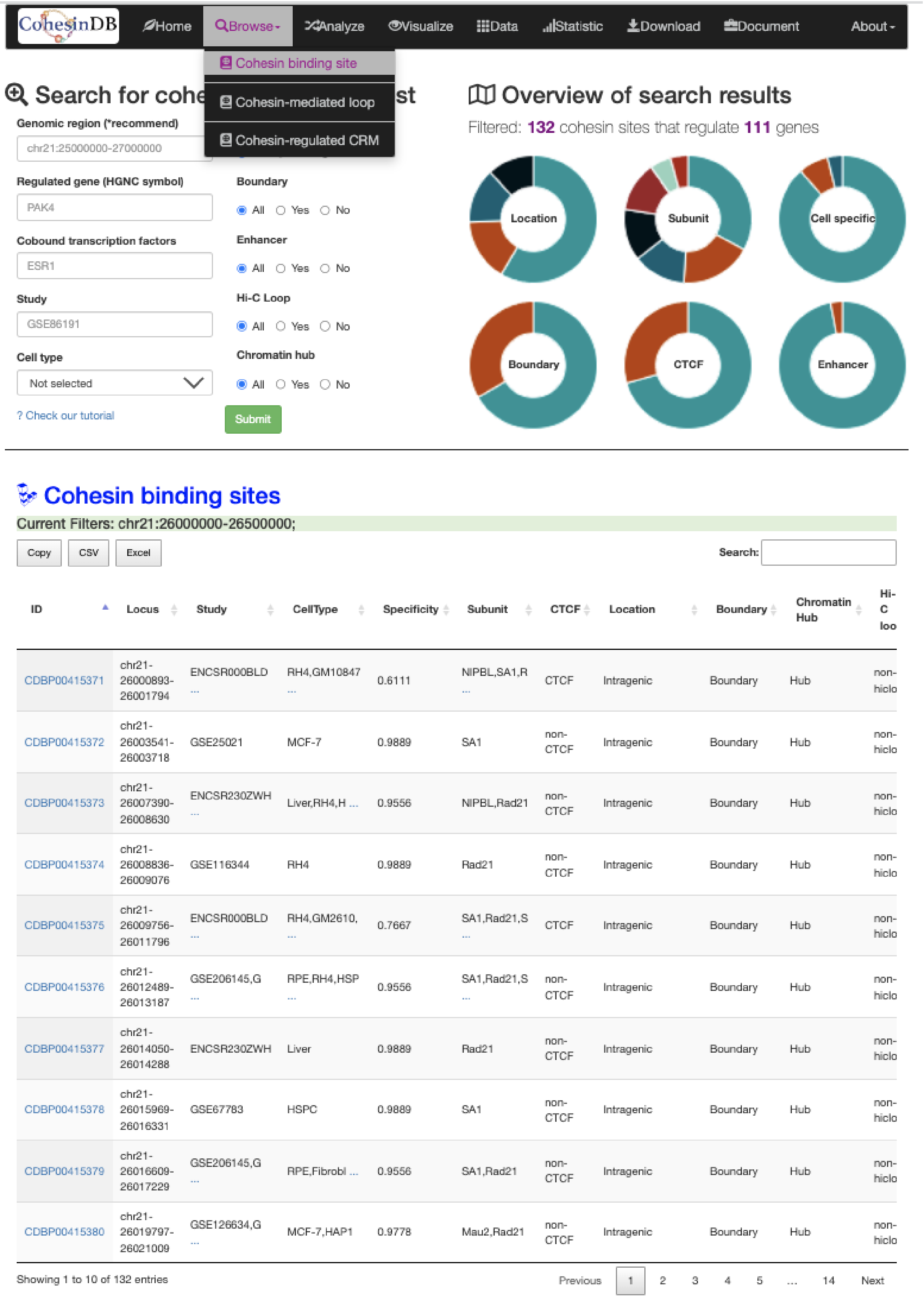


**Figure S5.** Screenshot of the “Browse” page for a cohesin binding object.

## Supplementary Figure S6

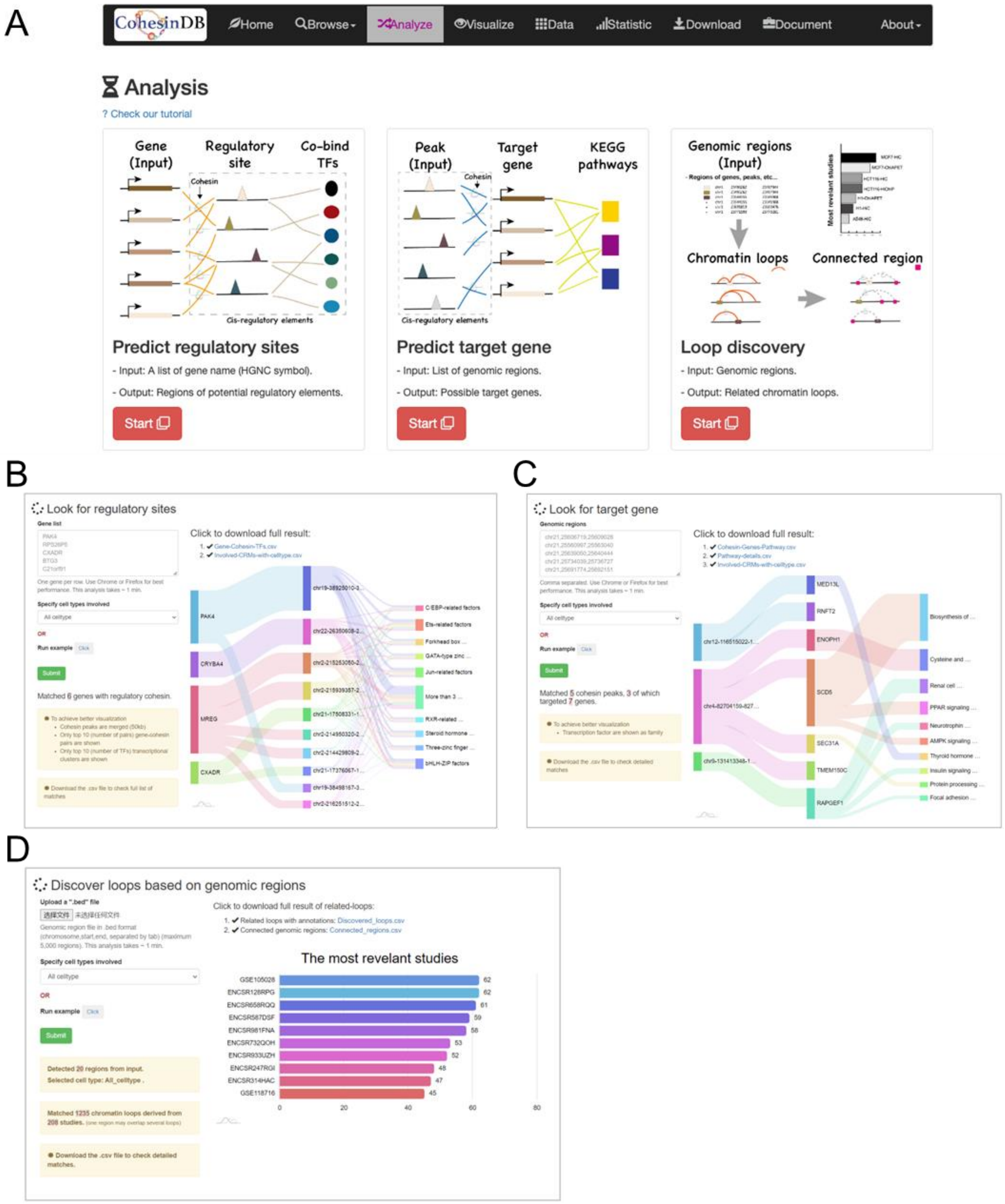


**Figure S6.** Screenshot of the "Analyze" page. (A) Users can choose one type of analysis. (B) Example of "predict regulatory sites". (C) Example of "predict target gene". (D) Example of "loop discovery".

# Supplementary Figure S7

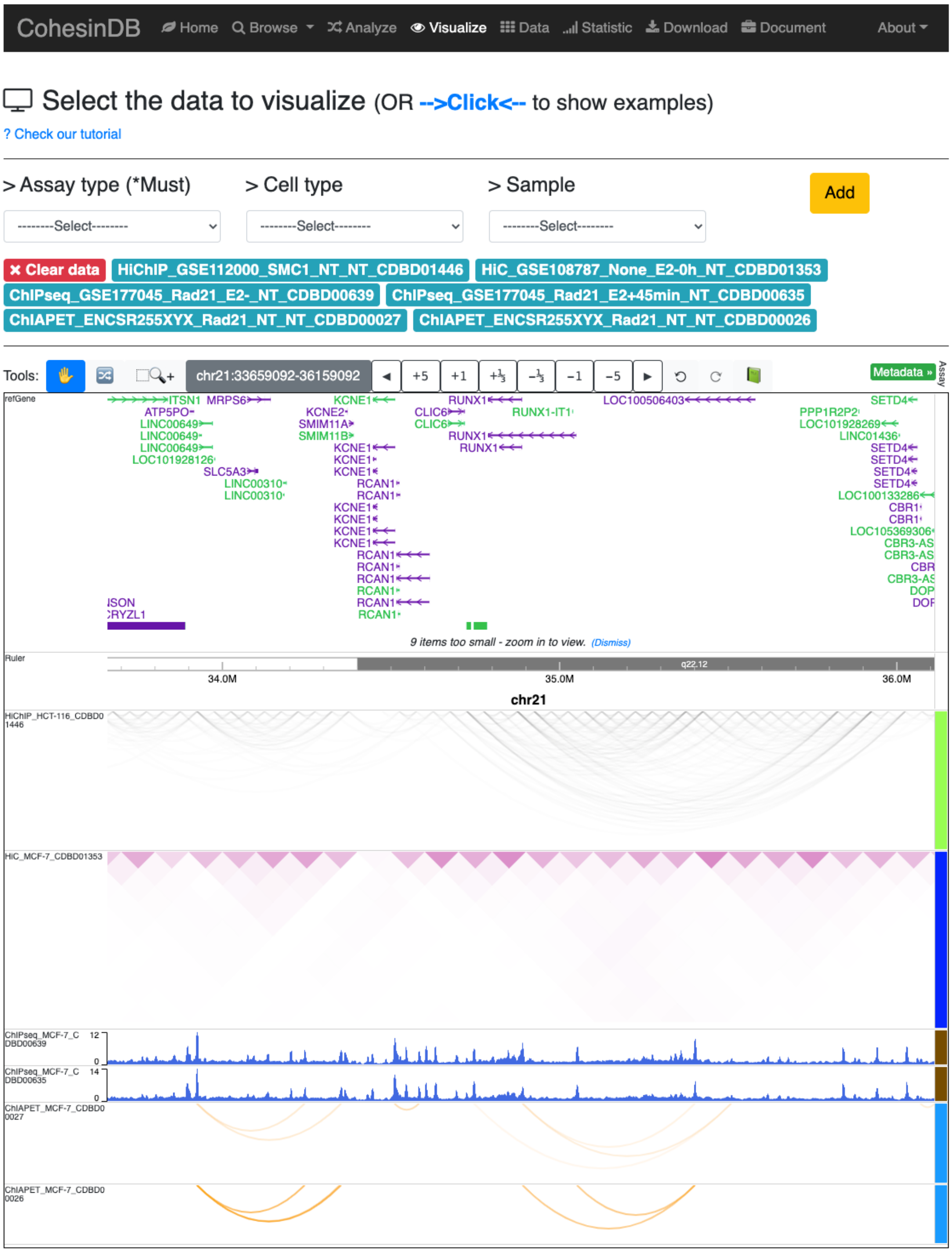


**Figure S7.** Screenshot of the "Visualize" page for processed data in CohesinDB.

## Supplementary Figure S8

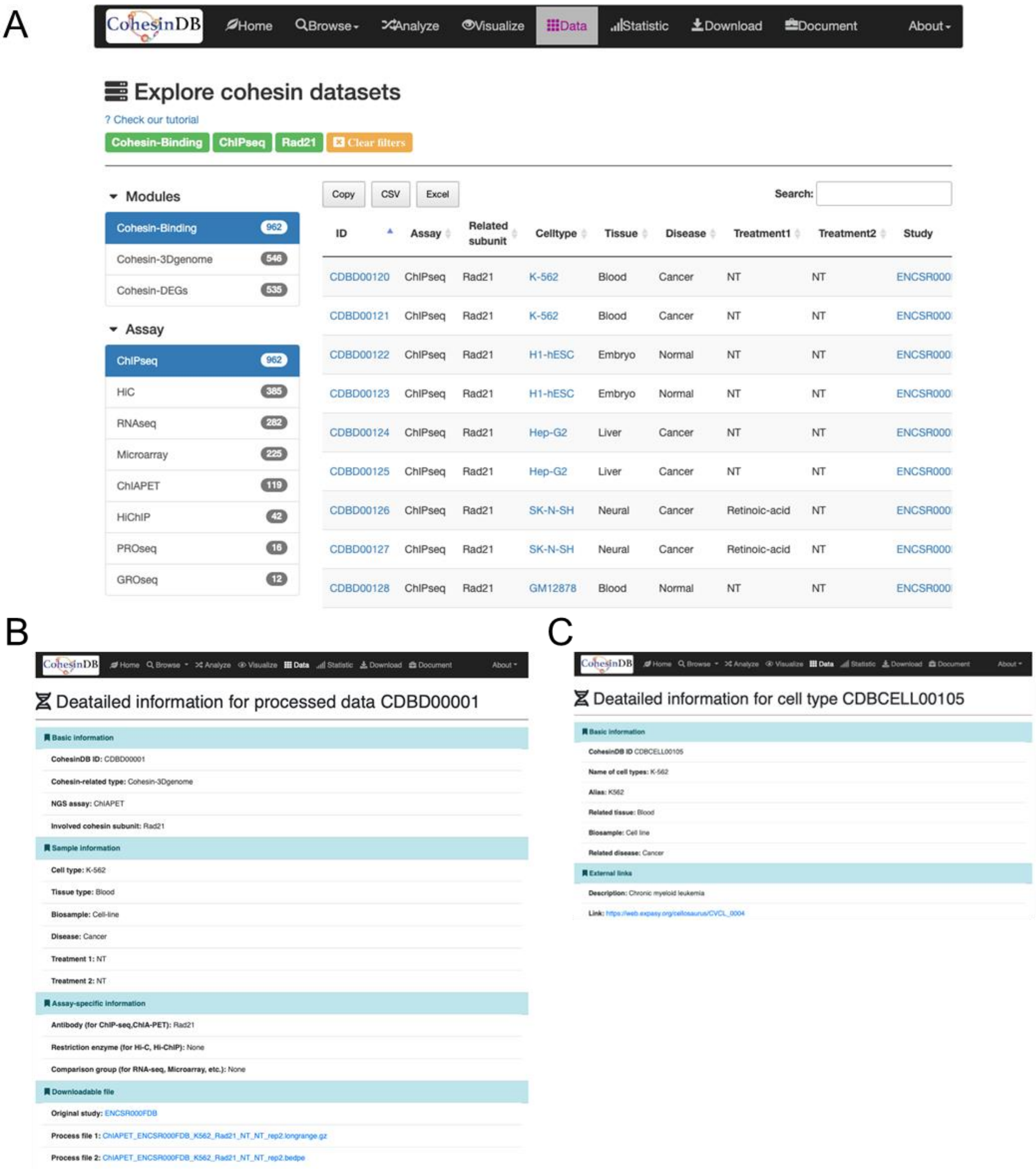


| ID | Assay | Related subunit | Celltype | Tissue | Disease | Treatment1 | Treatment2 | Study |
|---|---|---|---|---|---|---|---|---|
| CDBD00120 | ChIPseq | Rad21 | K-562 | Blood | Cancer | NT | NT | ENCSR000 |
| CDBD00121 | ChIPseq | Rad21 | K-562 | Blood | Cancer | NT | NT | ENCSR000 |
| CDBD00122 | ChIPseq | Rad21 | H1-hESC | Embryo | Normal | NT | NT | ENCSR000 |
| CDBD00123 | ChIPseq | Rad21 | H1-hESC | Embryo | Normal | NT | NT | ENCSR000 |
| CDBD00124 | ChIPseq | Rad21 | Hep-G2 | Liver | Cancer | NT | NT | ENCSR000 |
| CDBD00125 | ChIPseq | Rad21 | Hep-G2 | Liver | Cancer | NT | NT | ENCSR000 |
| CDBD00126 | ChIPseq | Rad21 | SK-N-SH | Neural | Cancer | Retinoic-acid | NT | ENCSR000 |
| CDBD00127 | ChIPseq | Rad21 | SK-N-SH | Neural | Cancer | Retinoic-acid | NT | ENCSR000 |
| CDBD00128 | ChIPseq | Rad21 | GM12878 | Blood | Normal | NT | NT | ENCSR000 |

**Figure S8.** Screenshot of the “Data” page. (A) Users can filter and browse the datasets of interest. (B) By clicking on the sample ID, users can check the detailed information of the processed data. (C) By clicking on the cell name, users can check the detailed information of cell types.

# Supplementary Figure S9

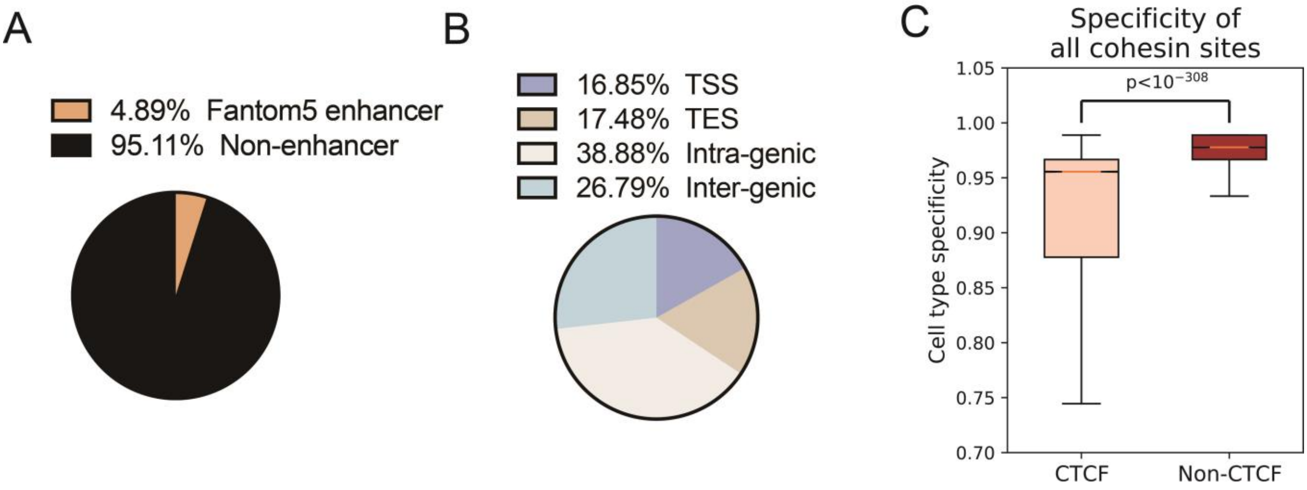


**Figure S9.** Examination of cohesin information in CohesinDB. (A) Percentage of cohesin sites annotated as enhancers based on the Famtom5 project. (B) Genomic distribution of all cohesin sites based on the RefSeq reference. (C) Cell-type specificity of CTCF cohesin and non-CTCF cohesin sites. A significant p value (Mann–Whitney U test) was observed.

# Supplementary Figure S10

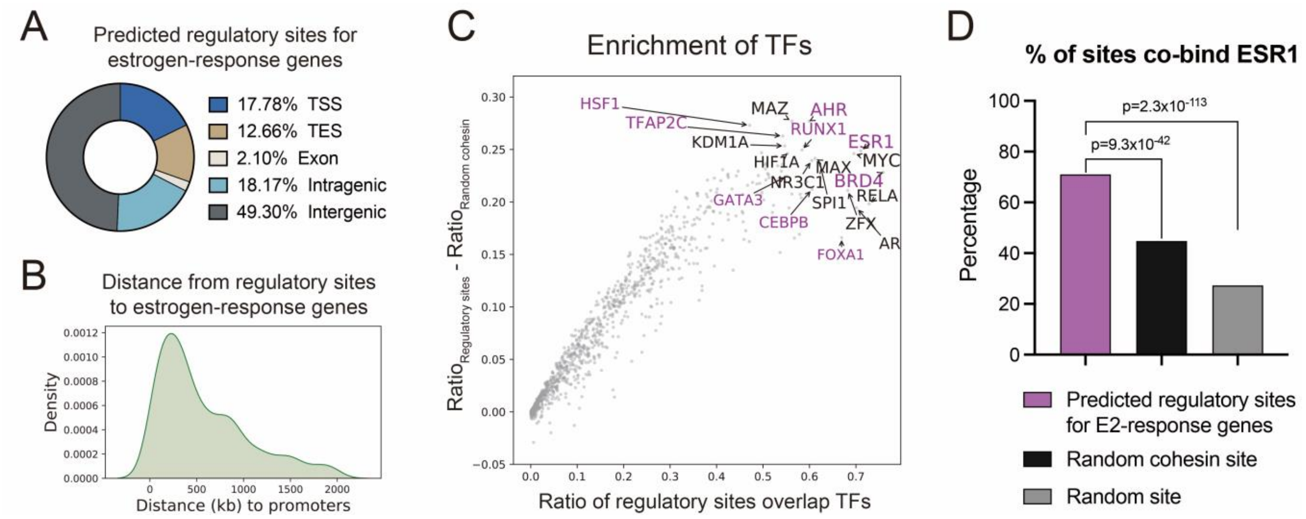


**Figure S10.** Analyzing estrogen response genes with CohesinDB. (A) Genomic distribution of predicted regulatory sites. (B) Distance from regulatory sites to promoters. (C) Enrichment of transcription factors. Read colors represent well-known TFs for estrogen response elements. (D) Percentage of sites co-binding ESR1. Fisher's exact test was used. Random sites were chosen from chromatin segmentations with a resolution of 1 kb.
